# In Mourning and Memory of Late Professor Zhou Jun

**DOI:** 10.1007/s13659-021-00300-5

**Published:** 2021-02-23

**Authors:** Ji-Kai Liu, Xiao-Dong Luo

**Affiliations:** 1 School of Pharmaceutical Science, South-Central University for Nationalities, Wuhan, People’s Republic of China; 2 State Key Laboratory of Phytochemistry and Plant Resources in West China, Kunming Institute of Botany, Chinese Academy of Sciences, Kunming, People’s Republic of China

Professor Zhou Jun, a world-renown natural products chemist, and resource botanist, passed away on March 27, 2020, at the age of 89 in Kunming, China. The natural products and plant resource research field lost a noble and outstanding leader when he died since he had contributed very much to the field in a variety of aspects. He was also one of the honorary editors-in-chief of *Natural Products and Bioprospecting*. We deeply mourn Professor Zhou’s loss and dedicate this note to him.

Professor Zhou was born on February 5, 1932, in Dongtai, Jiangsu Province, China. He started to study at the National College of Pharmacy in Nanjing in 1948. He was then educated in East China University of Chemical Technology from 1954 to 1958. After he graduated, he joined to Kunming Institute of Botany, the Chinese Academy of Sciences.

Professor Zhou was an active and accomplished scientist who worked on the national demands. He and his colleagues searched diosgenin, hecogenin, and tigogenin from domestic plants as new raw materials for the production of contraceptive drugs in China. He also developed the technique for the production of colchicine from *Iphigenia indica*. He found the active component gastrodin in *Gastrodia elata* and successfully developed the drug for migraine and neurasthenia therapy.

Professor Zhou was one of the pioneers in the Chinese phytochemistry society. He focused his research on plant saponins and cyclopeptides for many years. He published over 400 peer-reviewed scientific papers during his career, pushing forward the frontiers and developments of phytochemistry in China.

Through the years, Professor Zhou mentored numerous postdoctoral fellows, Ph. D., and graduate students pursuing careers in academia and various other industries. He was an excellent mentor, supportive and simultaneously firm. A long list of students and young scientists have, directly or indirectly, benefited from his expert knowledge and encouragement and followed in his footsteps. In this issue, some of them, as authors, are publishing articles dedicated to him.

Professor Zhou will be remembered as a significant pillar within the scientific and phytochemistry communities worldwide—we mourn his passing deeply.


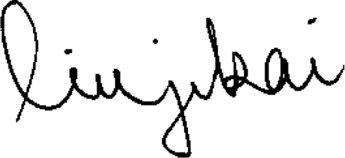


Prof. Dr. Liu Ji-Kai

Editor-in-Chief of *Natural Products & Bioprospecting*


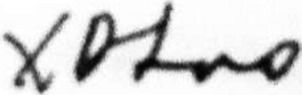


Prof. Dr. Luo Xiao-Dong

Executive Editor-in-Chief of *Natural Products & Bioprospecting*

